# Clinical outcomes of MII oocytes with refractile bodies in patients undergoing ICSI and single frozen embryo transfer

**DOI:** 10.1002/rmb2.12305

**Published:** 2019-11-25

**Authors:** Hiromi Takahashi, Junko Otsuki, Michio Yamamoto, Hiroe Saito, Rei Hirata, Toshihiro Habara, Nobuyoshi Hayashi

**Affiliations:** ^1^ Okayama Couple's Clinic Okayama Japan; ^2^ Assisted Reproductive Technology Center Okayama University Okayama Japan; ^3^ Graduate School of Environmental and Life Science Okayama University Okayama Japan

**Keywords:** blastocyst, cytoplasmic morphology, embryo implantation, ICSI, refractile body

## Abstract

**Purpose:**

This study aimed to analyze whether the presence of refractile bodies (RFs) negatively affects fertilization, embryo development, and/or implantation rates following intracytoplasmic sperm injection (ICSI).

**Methods:**

This retrospective embryo cohort study involved a total of 272 patients undergoing ICSI treatment of blastocyst cryopreservation.

**Results:**

In the study, no significant differences were found regarding 2PN formation rates between RF(+) (76.5%) and RF(−) oocytes (77.2%). However, the blastocyst formation rate on Day 5 in RF(+) oocytes was 45.8%, which was significantly lower than that of 52.2% in RF(−) oocytes (aOR 0.74, 95% CI 0.59‐0.93, *P* = .011). Implantation rates were also significantly lower in RF(+) oocytes (24.2%) as compared to RF(−) oocytes (42.2%) (aOR 0.46, 95% CI 0.26‐0.78, *P* = .005). Furthermore, the implantation rate of RF(+) oocytes (28.6%), when high‐quality blastocysts were transferred, was significantly lower than that of RF(−) oocytes (46.1%) (aOR 0.50, 95% CI 0.25‐0.96, *P* = .043).

**Conclusion:**

Our results suggest that oocytes with the presence of RFs have a lower potential for blastocyst development. Even when they develop into high‐quality blastocysts, the chances of implantation are reduced.

## Graphical abstract

Oocytes with the presence of RFs have a lower potential to develop into blastocysts, and even when they develop into high‐quality blastocysts, the chances of implantation are reduced.

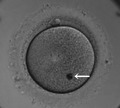

## 1 INTRODUCTION

**Figure 1:**
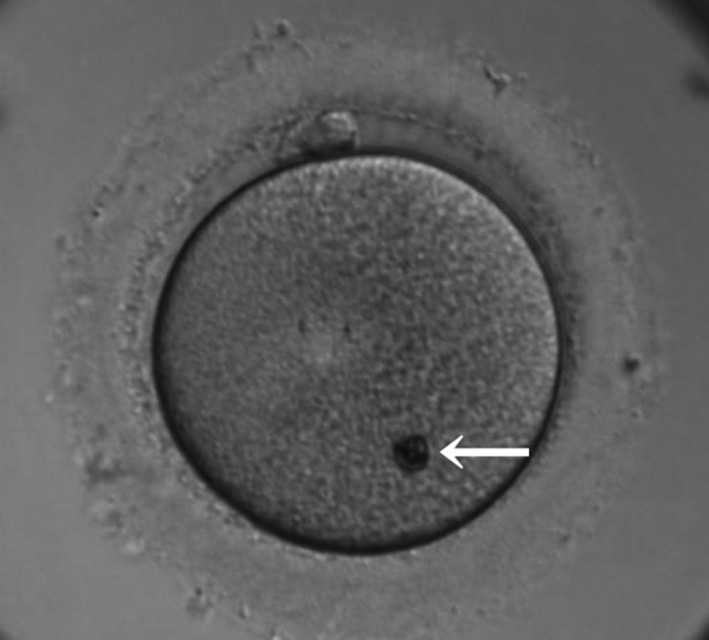
An image of a typical large refractile body (RF). An image of a typical large refractile body (RF) in a human MII oocyte. The refractile body is indicated by the arrow

A refractile body (RF) is one of the main morphological abnormalities, which can be observed in the cytoplasm of human oocytes (Figure 1). We previously found that RFs consist of a mixture of lipids and dense granular materials, have a yellow autofluorescence, which is consistent with the typical autofluorescence of lipofuscin, and have a positive reaction in the Schmorl reaction test, which demonstrates the presence of lipofuscin.^1^ Lower fertilization rates with IVF, but not ICSI, have also been reported.^1^ Lower embryo development rates 1, 2 of embryos with the presence of RFs have also been consistently reported. However, few reports have described any divergence in embryo quality between oocytes with or without RFs.^3^, ^4^ Furthermore, the implantation potential of oocytes with RFs has not yet been investigated. Thus, in this study we aimed to investigate whether RFs negatively affect embryo development and implantation rates following intracytoplasmic sperm injection (ICSI).

## 2 MATERIALS AND METHODS

This retrospective study included 316 RF(+) cycles, which had both RF(+) and RF(‐) oocytes, involving 272 patients who underwent ICSI treatment of blastocyst cryopreservation between January 2013 and June 2016. A total of 1190 blastocysts were cryopreserved, and of these, 438 were used for single embryo transfer by December 2016.

### 2.1 Stimulation protocols

The ovarian stimulation protocols were chosen depending on each patient's age and serum anti‐Müllerian hormone (AMH) level (Table 1). Patients in the long protocol group and the short protocol group were treated with the GnRH agonist (Nafarelil, Fuji Pharma), which continued until the day on which 10 000 IU of human chorionic gonadotropins (hCG) (HCG, Fuji Pharma) was administered. For the long protocol, administration of the GnRH agonist commenced a week after the previous ovulation. For the short protocol, administration of the GnRH agonist commenced on the 2nd day of the menstrual cycle. For the GnRH antagonist protocol, the GnRH antagonist ganirelix (Ganirest, Merck & Co) was administered when the leading follicle diameter reached 14‐15 mm in diameter and it was administered until the day of hCG administration. For the long, short, and antagonist protocols, ovarian stimulation began on the 3rd day of the cycle using hMG (Folyrmon‐P, Fuji Pharma; F, Fuji Pharma; HMG TEIZO, ASKA Pharmaceutical; Ferring, Ferring Pharmaceuticals) or rFSH (Gonalef, Merck Serono), and continued until the day of hCG administration. The starting dose for the clomiphene citrate (CC) protocol was 50 mg/day of (CC) (Clomid, Fuji Pharma) administered orally once a day, on the third day of the menstrual cycle, and continuing until the day of hCG administration. hMG was administered until the day of hCG administration. The starting dose for the aromatase inhibitor (AI) protocol was 5 mg/day of AI (Letrozole “TEVA,” Pharmaceutical Industries Ltd), administered orally on the third day of the menstrual cycle and continuing for 5 days. hMG was administered until the day of hCG administration. When at least two follicles reached a diameter of 18 mm or larger, 10 000 IU hCG was administered for ovulation induction.

**Table 1:** Ovarian stimulation protocols

AMH level (ng/mL)	<40 y	≧40 y
4< AMH	Antagonist, long	Antagonist, long
1< AMH ≦4	Long, antagonist	Antagonist, long, short
0.5< AMH ≦1	Short, antagonist, CC, AI	CC, AI, short, antagonist
0.1< AMH ≦0.5	CC, AI, short, antagonist	CC, AI
AMH ≦0.1	CC, AI	CC, AI, natural

### 2.2 Oocyte retrieval and denuding

Oocyte retrieval was performed 35.5 hours after hCG administration, with transvaginal ultrasound‐guided aspiration. Oocytes were collected from the follicular fluid and washed in freshly equilibrated human tubal fluid medium with HEPES (Multipurpose Handling Medium, Irvine Scientific). They were then incubated in an insemination medium (Universal IVF, Origio) covered with mineral oil (Light Mineral Oil for Embryo Culture) for 2‐4 hours with 6.5% CO_2_/5% O_2_/88.5% N_2_ at 37°C in an incubator MINC^TM^ (COOK MEDICAL INC). The oocytes were denuded of the cumulus oophorus by a brief exposure to 40 IU/mL hyaluronidase (ICSI Cumulase, Origio). This was followed by the mechanical removal of the oocytes from the surrounding cumulus cells with the use of a pipette (STRIPPER, Origio). Denuded oocytes were incubated in single‐step medium (ONE STEP Medium, NAKA ivf Medium) covered with mineral oil.

### 2.3 ICSI procedures and embryo culture

ICSI was performed on MII stage oocytes 4 hours after retrieval. MI stage oocytes were used for ICSI only when they reached MII stage maturity within 8 hours of oocyte retrieval (delayed MⅡ). At the time of ICSI, oocytes were morphologically evaluated under inverted microscopes (OLYMPUS) at ×400 magnification. MII oocytes with the presence of RF ≥5 µm in diameter were recorded as RF(+), and MII oocytes with the presence of RF <5 µm in diameter were recorded as RF(‐). This classification was based on our previous data, which showed that large RFs (>5 μm) were associated with significantly lower blastocyst development rates.^1^

The diameter of the (RFs) was measured by means of a scale bar displayed on a monitor connected to an OLYMPUS inverted microscope, at ×400 magnification. When the size of an RF was greater than the length of the scale bar, the RF was defined as RF(+). Sperm were prepared using a density‐gradient centrifugation technique with Isolate (Irvine, Cal., USA) and the swim‐up method with Universal IVF. Fertilization was confirmed by the presence of two pronuclei 16‐18 hours after ICSI (Day 1). The zygotes were placed into the well‐of‐the‐well (WOW) culture system (LinKID^®^ micro25, DNP) using a single‐step medium, and covered with mineral oil. Cleaving embryos were evaluated on the third day after oocyte retrieval using Veeck's classification.^5^ Blastocyst development was evaluated on the fifth and sixth days after oocyte retrieval by means of Gardner's classification.^6^ High‐quality blastocysts were defined as having a grade of at least 3BB on Day 5.

### 2.4 Vitrified‐thawed blastocyst transfer

The timing of cryopreservation depended entirely on blastocyst development. Blastocysts (grades 3‐6) with good/fair ICM‐TE morphology (AA, AB, AC, BA, BB, BC, CA, and CB) were selectively cryopreserved on Day 5, with the exception of those with poor ICM‐TE morphology (CC). Blastocysts not reaching this benchmark were given a further day in culture, after which they were cryopreserved with vitrification media (Cryotop, KITAZATO) on Day 6. Thawing procedures were performed with a thawing media (Cryotop, KITAZATO), and laser‐assisted hatching was performed immediately after thawing. Embryo transfers were carried out with a transfer medium (UTM^TM^, Origio), after 2‐3 hours of additional culture in the single‐step medium. Frozen‐thawed embryo transfers were performed with endometrial preparation by means of either the natural ovulation cycle or a cycle of hormone replacement therapy. All single embryo transfer procedures were performed under transabdominal ultrasound guidance, with the use of catheters for the embryo transfer (Wallace^®^ Sure‐Pro^®^, Smiths Medical). Pregnancy was confirmed by serum hCG concentration, 10 days after the embryo transfer. Clinical pregnancy was defined as the presence of a gestational sac, detected by ultrasound, while miscarriage was defined as the failed continuation of a pregnancy at under 22 weeks. Both miscarriages and stillbirths were considered as loss of pregnancy.

### 2.5 Statistical analyses

The differences in fertilization rates, blastocyst formation rates, and implantation rates were compared between RF(+) and RF(−) oocytes. The difference in fertilization rates was compared by the chi‐squared test. The difference in blastocyst formation rates, implantation rates, live birth rates, and pregnancy loss rates was investigated using a mixed‐effects logistic regression model (generalized linear mixed model, GLMM). GLMM provides a broad range of models for the analysis of grouped data, allowing the differences between groups to be modeled as random effects. Furthermore, as embryo transfer is repeated more than once for some patients, GLMM makes it possible to eliminate individual differences. The difference in blastocyst formation rates was investigated with a GLMM containing terms for RF(±) and patients' age as fixed effects, and patients as a random effect, taking inter‐subject correlation into consideration. Implantation, live birth, and pregnancy loss rates were analyzed with GLMM, containing terms for RF(±), age, and the presence of high‐quality blastocyst development (0/1) as fixed effects, and patients as a random effect, taking inter‐subject correlation into consideration. In all analyses, two‐sided statistical tests were performed, and differences were considered statistically significant when the *P*‐value was <.05. R software (version 3.4.4) was used for all statistical analyses.

## 3 RESULTS

The average age of participants was 37.8 years. A total of 3085 MⅡ oocytes were retrieved. Of these, 648 (21.0%) were RF(+) oocytes. There were 241 delayed MⅡ oocytes (7.8%), and the rate of RF(+) found among these oocytes was 16.6%. There was no significant difference between the occurrence of RFs in MⅡ oocytes and occurrence in delayed MⅡ oocytes (Table 2).

**Table 2:** The occurrence of RF according to type of oocyte and ovarian stimulation protocol

Ovarian stimulation protocol	RF(+) in matured MⅡ oocytes (95% CI)	RF(+) in delayed MⅡ oocytes (95% CI)	OR (95% CI)	*P*‐value
Total	21.4% (19.9‐22.9)	16.6% (12.4‐21.8)	0.73 (0.52‐1.04)	.073
Long	19.9% (17.3‐22.9)	16.9% (9.7‐27.8)	0.82 (0.42‐1.60)	.549
Short	26.1% (21.0‐31.9)	38.1% (20.8‐59.1)	1.74 (0.69‐4.39)	.250
Antagonist	18.1% (16.1‐20.4)	11.8% (7.1‐18.8)	0.60 (0.34‐1.07)	.067
Antagonist + AI	20.7% (16.4‐25.8)	8.7% (2.4‐26.8)	0.36 (0.08‐1.60)	.129
CC	36.8% (30.8‐43.1)	45.5% (21.3‐72.0)	1.43 (0.43‐4.84)	.564
AI	36.8% (23.4‐52.7)	0%	0	.182

Abbreviations: CI, confidence intervals; OR, odds ratio.

The data were analyzed by GLMM to clarify whether the stimulation protocol affected the outcomes and/or presence of RFs. No significant differences were found regarding 2PN formation rates between RF(+) (76.5%) and RF(−) (77.2%) oocytes. 1PN formation rates were 2.2% in RF(+) oocytes vs 2.4% in RF(−) oocytes, and no statistical differences were detected. In addition, multiple pronuclei formation (>3PN) rates were 2.2% in RF(+) oocytes vs 1.9% in RF(−) oocytes, with no significant differences being detected (Table 3). However, the blastocyst formation rate on Day 5 in RF(+) oocytes was 45.8%, which was significantly lower than that of 52.2% in RF(−) oocytes (aOR 0.74, 95% CI 0.59‐0.93, *P* = .011; Table 3). The rate of blastocysts that reached blastocysts on Day 6 for RF(+) was 10.5%, while it was 9.6% for RF(−) oocytes. No significant differences were found between the two groups (aOR 1.11; 95% CI 0.79‐1.56, *P* = .536; Table 3). The rate of Day 6 blastocysts that were transferred during vitrified‐thawed blastocyst transfer cycles was 20.2% for RF(+) and 13.9% for RF(−) oocytes, respectively. No significant differences were detected between the two groups (OR 1.57, 95% CI 0.87‐2.76, *P* = .125). Implantation rates were significantly lower in RF(+) oocytes (24.2%) as compared to RF(−) oocytes (42.2%) (aOR 0.46, 95% CI 0.26‐0.78, *P* = .005). Furthermore, the implantation rate in RF(+) oocytes (28.6%) when high‐quality blastocysts were transferred was significantly lower than that in RF(−) oocytes (46.1%) (aOR 0.50, 95% CI 0.25‐0.96, *P* = .043; Table 4). However, no significant differences were found regarding pregnancy loss rates (20.8% in RF(+), 30.8% in RF(−) oocytes, aOR 0.57; 95% CI 0.17‐1.59, *P* = .318; Table 4). Neither were any significant differences detected in the birth rates (19.2% in RF(+), 29.2% in RF(−) oocytes, aOR 0.60, 95% CI 0.31‐1.08, *P* = .101; Table 4).

**Table 3:** Clinical outcomes of embryo development according to type of oocyte

	RF(‐)oocytes		RF(+)oocytes		Crude difference					Adjusted difference				
		95% CI		95% CI	Estimated Regression Coefficient	SE	COR	95% CI	*P*‐value	Estimated Regression Coefficient	SE	aOR	95% CI	*P‐*value
N. of oocytes	2437		648											
2PN	77.2% (1881/2437)	75.5‐78.8	76.5% (496/648)	73.1‐79.6	−0.036	0.105	0.96	0.79‐1.19	.730					
1PN	2.4% (59/2437)	1.9‐3.1	2.2% (14/648)	1.3‐3.6	−0.117	0.301	0.89	0.47‐1.56	.698					
>3PN	1.9% (46/2437)	1.4‐2.5	2.2% (14/648)	1.3‐3.6	0.138	0.309	1.15	0.60‐2.05	.655					
No. of embryos	1881		496											
Blastocyst formation rate on Day 5^a^	52.2% (982/1881)	49.9‐54.5	45.8% (227/496)	41.4‐50.2	−0.258	0.101	0.77	0.63‐0.94	.011	−0.297	0.116	0.74	0.59‐0.93	.011
Blastocyst formation rate on Day 6	9.6% (181/1881)	8.3‐11.0	10.5% (52/496)	7.9‐13.5	0.095	0.166	1.10	0.79‐1.51	.566	0.108	0.174	1.11	0.79‐1.56	.536
High‐quality blastocyst formation rate^a^	24.0% (451/1881)	22.1‐26.0	20.2% (100/496)	16.9‐23.9	−0.222	0.124	0.80	0.63‐1.02	.069	−2.032	0.139	0.82	0.62‐1.07	.144

Abbreviations: aOR, adjusted odds ratio; CI, confidence interval; COR, crude odds ratio.

a Blastocyst formation rates and high‐quality blastocyst formation rates were analyzed using a mixed‐effects logistic regression model that contains terms for patients' age and the presence of RFs (0/1) as fixed effects and patient‐specific intercept as a random effect.

**Table 4:** Clinical outcomes of implantation potential according to type of oocytes

	RF(‐) oocytes		RF(+) oocytes		Crude difference					Adjusted difference				
		95% CI		95% CI	Estimated regression coefficient	SE	COR	95% CI	*P*‐value	estimated regression coefficient	SE	aOR	95% CI	*P*‐value
No. of transferred embryos	339		99											
Implantation rate	42.2% (143/339)	37.0‐47.5	24.2% (24/99)	16.9‐33.5	−0.824	0.259	0.44	0.26‐0.72	.001	−0.778	0.278	0.46	0.26‐0.78	.005
Implantation rate of high‐quality blastocyst transferred	46.1% (111/241)	39.9‐52.4	28.6% (18/63)	18.9‐40.7	−0.758	0.307	0.47	0.25‐0.84	.014	−0.688	0.341	0.50	0.25‐0.96	.043
Live birth rate	29.2% (99/339)	24.6‐34.3	19.2% (19/99)	12.6‐28.0	−0.552	0.282	0.58	0.32‐0.98	.050	−0.514	0.314	0.60	0.31‐1.08	.101
Live birth rate of high‐quality blastocyst transferred	33.6% (81/241)	27.9‐39.8	22.2% (14/63)	13.7‐33.9	−0.572	0.332	0.56	0.29‐1.06	.085	−0.523	0.378	0.59	0.27‐1.21	.166
aNeonatal abnormality rate	3.0% (3/99)	1.0‐8.5	0% (0/19)						.995					
Pregnancy loss rate	30.8% (44/143)	23.8‐38.8	20.8%^b^ (5/24)	9.2‐40.5	−0.524	0.534	0.59	0.19‐1.58	.327	−0.557	0.558	0.57	0.17‐1.59	.318
Pregnancy loss of high‐quality blastocyst transferred	27.0% (30/111)	19.6‐36.0	22.2% (4/18)	9.0‐45.2	−0.260	0.606	0.77	0.21‐2.35	.668	−0.277	0.619	0.76	0.19‐2.41	.655

Note

Implantation rates, live birth rates, and pregnancy loss rates were analyzed using a mixed‐effects logistic regression model that contains terms for the presence of RFs (0/1), age, and high‐grade blastocyst development (0/1) as fixed effects and patient‐specific intercept as a random effect.

Abbreviations: aOR, adjusted odds ratio; CI, confidence interval; COR, crude odds ratio.

a The neonatal abnormalities found in the newborn babies were pulmonary valve stenosis, small for gestational age, and cleft palate.

b One stillbirth, occurring at 24 wk and 6 d, has been included in the pregnancy loss rate.

## 4 DISCUSSION

With this study, we revealed that oocytes with the presence of RFs have a lower potential to develop into blastocysts and that even when they do develop into high‐quality blastocysts, the chances of implantation are reduced. With regard to fertilization rates, no significant differences were detected between RF(+) and RF(−) oocytes. Several studies have demonstrated that oocytes with the presence of RFs impair fertilization by IVF.^1^, ^5^, ^7^ However, our results demonstrate that the potential for fertilization is not negatively affected by the use of ICSI. As previously described by Otsuki et al,^8^ the variance between RF(+) and RF(−) oocytes may account for some of the differences arising between fertilization rates with IVF and ICSI. The blastocyst formation rates of RF(+) oocytes on Day 5 were significantly lower than those of RF(−) oocytes. This was consistent with the data reported by Xia et al,^9^ demonstrating that the presence of RFs correlates strongly with poor embryo quality. Interestingly, although oocytes with the presence of RFs have been associated with significantly lower implantation rates than oocytes without the presence of RFs, even when high‐quality blastocysts are transferred, no significant difference was detected in the miscarriage or live birth rates in this study. This may suggest that the presence of RFs negatively affects the implantation process. As it has been reported that patients with severe endometriosis display a significantly higher proportion of RFs in their oocytes, the association of this tendency with the lower implantation rates found in our study should be further investigated in a future study. This would further elucidate whether the presence of RFs itself lowers the implantation potential or whether other molecular factors caused by endometriosis result in the impairment of embryo implantation.^10^

Regarding the outcome of in vitro–matured oocytes, there was no significant difference between the occurrence of RFs in matured oocytes (MⅡ) vs the occurrence found in oocytes with delayed maturation (delayed MⅡ). Conversely, Omidi et al^11^ have reported that the occurrence of RFs was significantly higher in IVM MII oocytes, which were matured from GV and MI oocytes, as compared to matured MII oocytes. The subject of our study was oocytes, which had reached MⅡ stage maturity within 8 hours of oocyte retrieval. RFs were detected in both mature and immature oocytes,^1^, ^5^ and there was a strong tendency for recurrence in the same patient in cases of repeat treatment^5^; ergo, the development of RFs may occur before or during folliculogenesis.

As this study did not extend to oocytes with single or multiple RFs, it is still unclear whether the size and number of RFs influence embryo development and/or implantation rates. The occurrence mechanisms of RFs and their relationship to oocyte maturation and viability are also not yet fully understood. Further research is required to gain a more complete understanding of these factors and thus improve implantation rates.

## CONFLICT OF INTEREST

Hiromi Takahashi, Junko Otsuki, Michio Yamamoto, Hiroe Saito, Rei Hirata, Toshihiro Habara, and Nobuyoshi Hayashi declare that they have no conflicts of interest.

## Human/Animal rights

This article does not contain any experimental studies with human or animal participants on the part of any of the authors. This study received the approval of the institutional review board of Okayama Couple's Clinic (Approval No.: 18000128‐13).
